# Follow-up of hereditary endometrial carcinoma caused by *MLH3* gene mutation: a case report

**DOI:** 10.3389/fonc.2025.1532908

**Published:** 2025-04-28

**Authors:** Changlin Zhang, Jiaying Ye, Qiaqia Li, Jijie Zhan, Dong Yang, Jundong Li, Tian Li, Ting Wan

**Affiliations:** ^1^ Department of Gynecological Oncology, Sun Yat-Sen University Cancer Center, Guangzhou, China; ^2^ Department of Gynecology, The Seventh Affiliated Hospital of Sun Yat-Sen University, Shenzhen, China

**Keywords:** MLH3, endometrial cancer, cancer susceptibility, germline gene, case report

## Abstract

**Background:**

Endometrial cancer is a common cancer in women, partially linked to defects in mismatch repair function. Besides the well-known mismatch repair proteins, the *MLH3* gene may also contribute to cancer susceptibility.

**Case presentation:**

In this case report, we reported that two related mothers and daughters had mutations in some of their germline genes, with *MLH3* as a possible low-risk gene for endometrial cancer, which we further explored as contributing to the development of endometrial cancer.

**Conclusions:**

This case identifies germline heterozygous mutations in two patients, suggesting a potential role for *MLH3* in endometrial carcinogenesis, which may act as a low-risk factor to increase the risk of tumor susceptibility and does not rule out the possibility of synergistic increases in pathogenicity with other genes.

## 1 Introduction

Endometrial cancer is the sixth most common cancer in women, with 420,245 new cases and 97,704 deaths globally in 2022. Its incidence has risen by 132% over the past 30 years, with the highest rates reported in North America (1). By 2022, China reported 77,722 cases of endometrial cancer and 13,511 deaths. Over 90% of patients are older than 50, with most people finding out they have it around age 63 (2). And only 4% are younger than 40. About 5% of these cancers are connected to genes passed down in families - the most common one being Lynch syndrome, a hereditary condition that increases cancer risk (3). Genetically, endometrial cancer is linked to defects in the mismatch repair system, involving two main protein families: MutS (*MSH2, MSH3, MSH6*) and MutL (*MLH1, MLH3, PMS1, PMS2*). Germline mutations in *MLH1, MSH2, MSH6*, and *PMS2*, which are dominantly inherited, lead to Lynch syndrome and endometrial cancer (4). The second reason for defective mismatch repair function is the methylation of the promoter region of genes encoding mismatch repair proteins, particularly *MLH1*, resulting in epigenetic alterations (5). In addition to major mismatch repair genes, *MLH3*, a newer member of the DNA mismatch repair family, also contributes to tumor development (6). But in the screening of colorectal cancer patients, tumors harboring *MLH3* mutations were identified as microsatellite-stable, suggesting that *MLH3* does not promote carcinogenesis through classical DNA mismatch repair deficiency but may act via alternative pathways (7). Building upon these findings, we utilized two representative endometrial carcinoma cases to investigate the role of *MLH3* in the pathogenesis and progression of endometrial cancer, with the aim of further elucidating its molecular mechanisms in oncogenic processes.

This study presents two cases of endometrial cancer to investigate the impact of germline mutations in *MLH3* on carcinogenesis and its mechanisms, potentially broadening the scope of genetic testing for endometrial cancer.

## 2 Cases presentation

### 2.1 The presentation of the patient’s diagnosis and treatment

A 50 years old Asian woman experienced abdominal pain for six months, with a blood test in July 2019 indicating a slightly elevated CA199 level. In May 2020, a Color Doppler ultrasound revealed a large mass in the abdominal and pelvic cavities, suspected by MRI as a mucinous cystadenocarcinoma of ovarian origin. The patient had no menstrual changes or abnormal bleeding.

**Figure 1:**
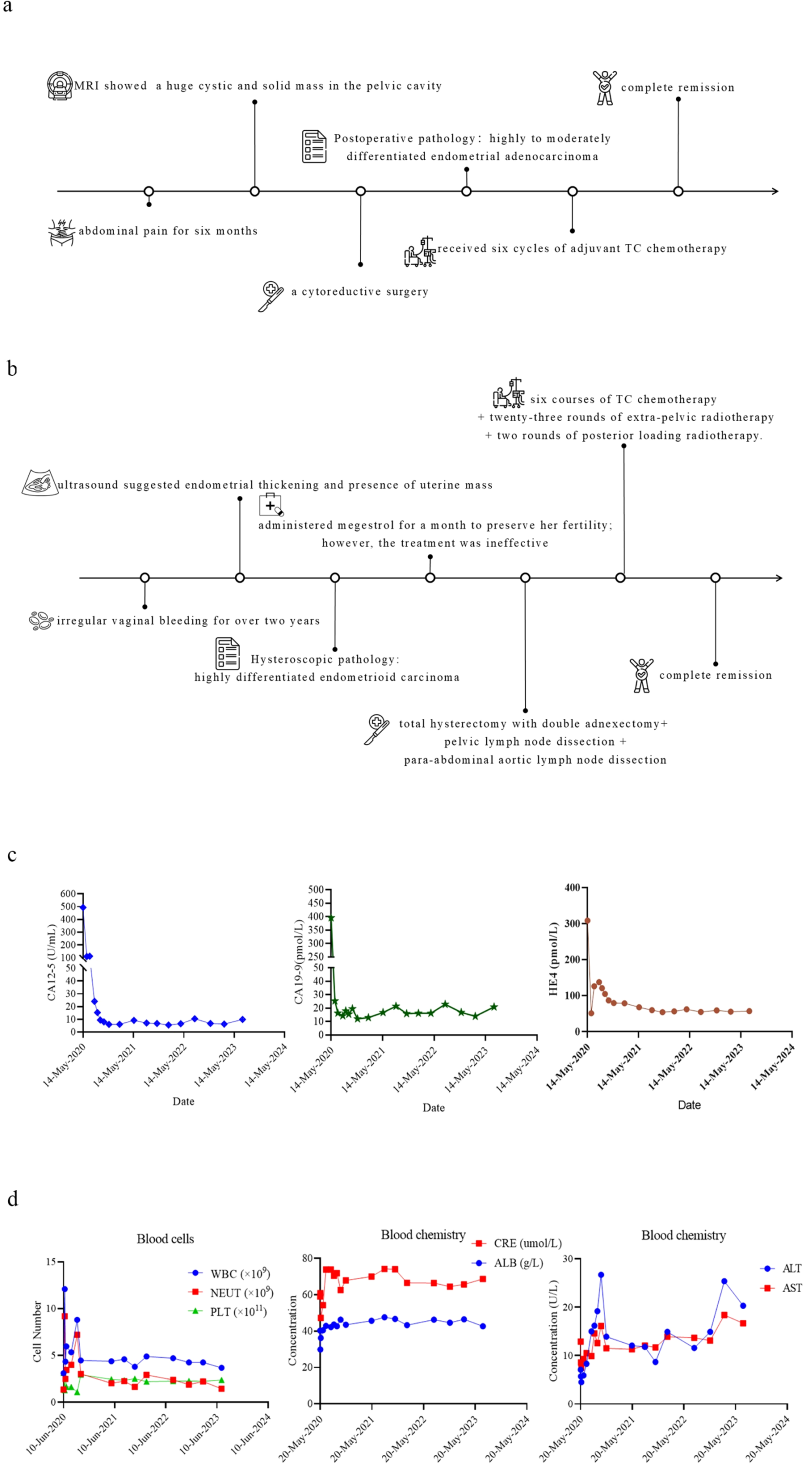
Information for patients. **(a)**, Important patient-related events. **(b)**, Important events related to the patient’s daughter. **(c)**, The expression levels of the cancer biomarkers during treatment. **(d)**, The dynamic levels of blood cells and blood chemistry during treatment. (A) Blood cells: WBC, NEUT, and PLT. (B) Blood chemistry: CRE and ALB. (C) Blood chemistry: ALT and AST.

She underwent a comprehensive surgery, including total hysterectomy with bilateral adnexectomy, omentectomy and lymph node evaluation. The left ovarian tumor, measuring 15 × 13 × 12 cm, was cystic and solid with sticky yellow mucus. Pathological examination revealed ovarian adenocarcinoma, with immunohistochemistry showing positive Estrogen Receptor (90%), Progesterone Receptor (40%), and CA125(+). Special staining indicated moderately differentiated endometrioid adenocarcinoma, with no cancer involved in lymph nodes or the omentum. Endometrial findings showed complicated atypical hyperplasia and highly differentiated endometrial cancer, and according to the 2009 version of the FIGO staging system for endometrial cancer, this patient was diagnosed with stage IIIA endometrial cancer. Important patient-related events are shown in **Figure 1a**.

Therefore, after surgery, the patient received six cycles of adjuvant chemotherapy (Abraxane 200–250 mg/m^2^, Carboplatin AUC=5). Regular CT scans post-chemotherapy revealed no recurrent or metastatic lesions with normal serum tumor markers. Thus, effectiveness was assessed as complete remission after six cycles of chemotherapy. The CA125, HE4, and CA199 levels of the patient remained normal and stable after 3 years of treatment, as shown in **Figure 1c**. Moreover, the patient’s hematological system, liver function, and kidney function were not significantly impaired during chemotherapy treatment, as shown in **Figure 1d**.

### 2.2 The presentation of her daughter’s diagnosis and treatment

The patient’s daughter was diagnosed with endometrial carcinoma at age 30, presenting with irregular vaginal bleeding for over two years. In April 2022, ultrasound revealed endometrial thickening, a 2 cm left adnexal cystic mass, and a 3 cm right adnexal mass. Hysteroscopy showed a hard nodule in the lower uterine cavity with tiny polyps, and pathology confirmed complex hyperplasia and highly differentiated endometrioid carcinoma. PET-CT indicated metabolically active uterine lesions and suspicious lymph node metastasis.

To preserve her fertility, she was treated with megestrol for one month, but her CA125 and CA199 levels increased, and MRI indicated lymph nodes metastasis, prompting a shift to surgical intervention. On May 25, 2022, she underwent total hysterectomy with bilateral adnexectomy, omentectomy and lymph node dissection. Postoperative pathology revealed highly differentiated endometrial cancer with para-aortic lymph nodes metastasis. She was diagnosed endometrial cancer stage IIIC_2_ and subsequently received six cycles of chemotherapy (paclitaxal and carboplatin), combined with external beam radiotherapy, and brachytherapy, achieving complete remission confirmed by CT scans and normal tumor marker levels. Important events related to the patient’s daughter are shown in **Figure 1b**.

### 2.3 The presentation of genetic mutations (Genetic alterations)

**Figure 2:**
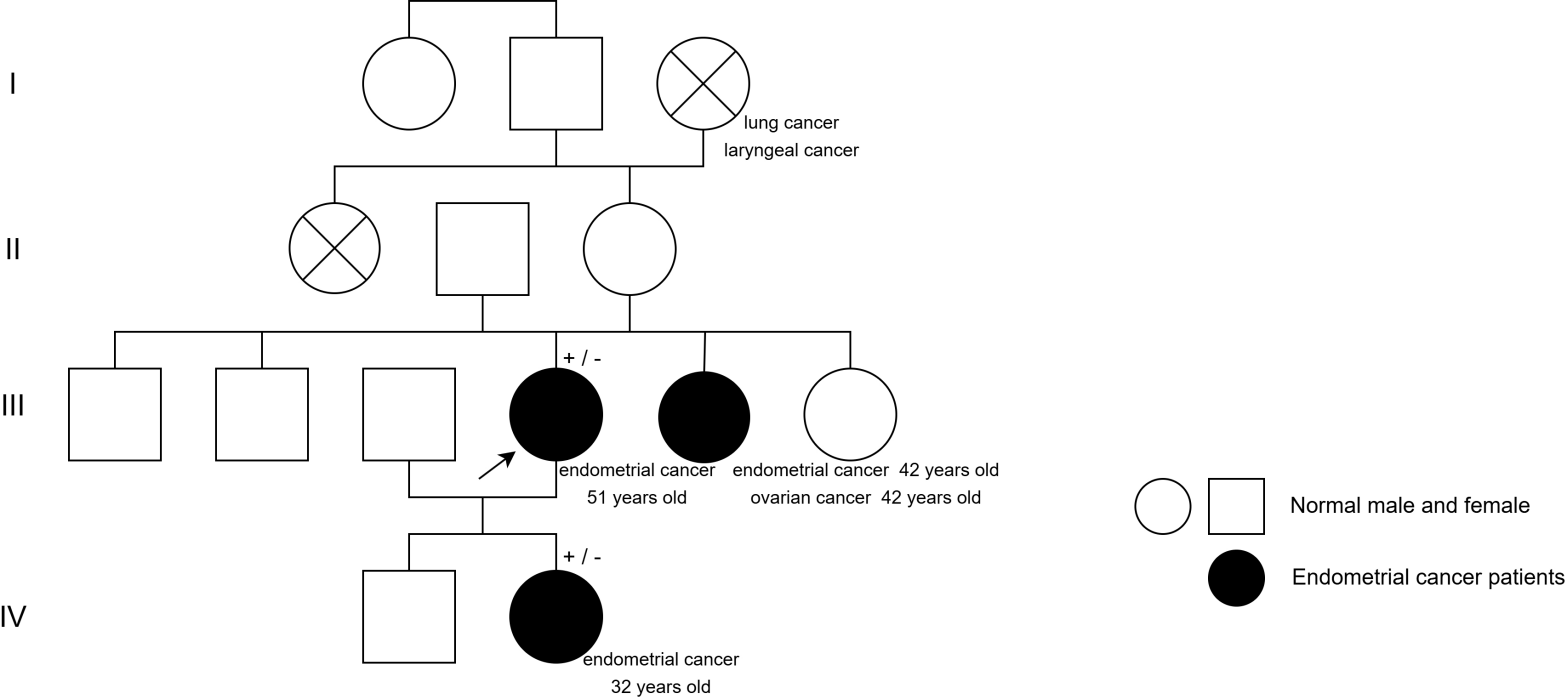
Genetic pedigrees.

Both the patient and her daughter had endometrial cancer, with the daughter diagnosed at a young age, indicating a potential family history of cancer. The patient’s younger sister had both endometrial and ovarian cancer (**Figure 2**). They were advised to undergo genetic testing to identify susceptibility genes for treatment guidance. Since the patient’s sister was diagnosed earlier, genetic screening was not prevalent at that time, so she did not undergo testing in this area. The patient’s testing for genitourinary tumor susceptibility genes revealed several mutations of uncertain significance (**Table 1**, **Supplementary Table 1**). Though reported in studies, their link to cancer remains unclear.

**Table 1:** The germline mutations of the patient.

Gene	Mutation	Location	Transcript	Homozygous/ Heterozygous	Functional change	Effect
*NBN*	c,1023C>G (p.Ser341Arg)	EX9	NM_002485.4	Heterozygous	Missense	unspecified significance
*MLH3*	c.3241G>C (p.Asp1081His)	EX2	NM_014381.2	Heterozygous	Missense	unspecified significance
*CHEK2*	c.613A>T (p.Thr205Ser)	EX5	NM_007194.3	Heterozygous	Missense	unspecified significance
*ATR*	c.2806-19A>G	IN13	NM_001184.3	Heterozygous	Splice(likely benign)	unspecified significance
*RBBP8*	c.249-3T>C	IN4	NM_203291.1	Heterozygous	Splice	unspecified significance
*SLX4*	c.2854_2855delinsAT (p.Ala952Met)	CDS11	NM_032444.3	Heterozygous	Missense	unspecified significance

The patient’s daughter underwent genetic testing for uterine tumors, which revealed several genetic mutations of unknown significance (**Tables 2**, **3**, **Supplementary Table 2**). Her tumor mutation burden was 1.43 Muts/Mb, with stable microsatellites and no mutations in key genes like *POLE* or *TP53*. Genetic testing revealed no specific molecular profile endometrial cancer in the daughter. Three germline mutations were present in both the patient and her daughter: *CHEK2* c.613A>T (p.Thr205Ser), *MLH3* c.3241G>C (p.Asp1081His), and *NBN* c.1023C>G (p.Ser341Arg). The specific germline mutant genes in the patient and the daughter are shown in **Tables 1**-**3**.

**Table 2:** The somatic mutations of the patient’s daughter.

Gene	Mutation	Location	Transcript	Mutation abundance	Variant class
*PTEN*	p.L146Ffs*34 (c.437dupT)	EX5	NM_000314.4	15.7%	II
*PTEN*	P.Y336*(c.1008C>G)	EX8	NM_000314.4	15.36%	II
*FBXW7*	p.R393*(c.1177C>T)	EX8	NM 033632.3	15.07%	II
*PIK3RI*	pI571Nfs*31 (c.1711dupA)	EX13	NM_181523.2	8.43%	II
*PIK3RI*	p.L570P (c.1709T>C)	EX13	NM_181523 2	16.57%	III
*PARP2*	p.L358V (c.1072T>G)	EX11	NM_005484.3	16.16%	III
*NUDTI8*	p.D222Y (c.664G>T)	EX3E	NM_024815.3	15.4%	III
*CDC42*	p.D11N (c.3G>A)	EX2	NM_001791.3	1.23%	III
*GRIN2A*	p.R1022C (c.3064C>T)	EX14E	NM_000833.3	0.7%	III
*FYN*	pK505Rfs*53 (c.1514delA)	EX14E	NM_002037.5	0.66%	III

**Table 3:** The germline mutations of the patient’s daughter.

Gene	Mutation	Location	Transcript	Homozygous/ Heterozygous	Inheritance patterns	Effect
*NBN*	c,1023C>G (p.Ser341Arg)	EX9	NM_002485.4	Heterozygous	Autosomal dominant(AD)	unspecified significance
*MLH3*	c.3241G>C (p.Asp1081His)	EX2	NM_014381.2	Heterozygous	AD	unspecified significance
*MET*	c.1871G>T (p.Cys624Phe)	EX7	NM_001127500.2	Heterozygous	AD	unspecified significance
*CHEK2*	c.613A>T (p.Thr205Ser)	EX5	NM_007194.3	Heterozygous	AD	unspecified significance
*CDKN1B*	x.*9-19G>A	IN2	NM_004064.4	Heterozygous	AD	unspecified significance

Previous literature reported these loci, but their pathogenicity remained unclear. Protein function prediction tools (SIFT, Polyphen-2, Mutation Taster) indicated that the *CHEK2* gene mutation was harmless, while the other two mutations were deleterious. Mutations in the *MLH3* gene cause DNA mismatch repair defects, increasing mutation rates and preventing apoptosis in severely damaged cells. Although functional redundancy of *MLH3* with *PMS2* can cause interaction with *MLH1* leading to defective DNA mismatch repair (8), this patient and her daughter showed only *MLH3* germline mutations and were both negative for microsatellite instability. Ruling out mismatch repair leading to endometrial cancer, we found that *MLH3* mutations are predominantly present in colorectal cancer patients in low-risk families, such as those in which two first-degree relatives have the disease, and that most of them are genetically inherited at low epistasis rather than a single highly pathogenic mutation. Similar to the mother and daughter in this case, as a microsatellite-stable *MLH3* mutant cancer population, germline mutations lead to increased tumor susceptibility in patients, and the existence of synergistic effects with other mutations causing endometrial cancer cannot be excluded.

## 3 Discussion

This study presents two related patients with concurrent endometrial cancer. Genetic testing revealed heterozygous germline mutations in *CHEK2*, *NBN*, and *MLH3*. The mutations in these patients are rare, making this case a novel contribution to our understanding of endometrial cancer germline mutations. Further investigation is needed to determine the pathogenicity and mechanisms of these mutations.

The *CHEK2* mutation is linked to colorectal and prostate cancer, but its association with endometrial cancer is unclear, and *CHEK2* is involved in DNA double-strand break repair, a dysregulated response may lead to tumorigenesis.

The *NBN* mutation is involved in cell cycle regulation and DNA damage repair, with carriers at increased risk for tumors, including CNS relapse of B-cell precursor acute lymphoblastic leukemia (9). The pathogenicity of the *NBN* and *CHEK2* mutations in this case remains unclear, and current literature has not specifically linked these mutations to endometrial cancer risk. this report examines the effect of *MLH3* gene mutations on the development of endometrial cancer.

*MLH3* is a core member of the DNA mismatch repair system and belongs to the MutL protein family. Its functions include: i) participation in the repair of DNA replication errors and maintenance of microsatellite stability; ii) participation in meiosis 1 crossover regulation and maintenance of germ cell stability; and iii) participation in alkylation damage and reduction of mutation accumulation induced by environmental carcinogens.The presence of malignant pathogenic mutations in *MLH3*, e.g., by interfering with the normal functioning of the *MLH1-MLH3* complex, induces dominant-negative effects that lead to MSI and cancer susceptibility. Some missense mutations or consent mutations with no significant effect also exist and do not affect the functional expression of the *MLH3* gene. However, in some atypical HNPCC —— hereditary type 7 colorectal cancer, *MLH3* germline mutations show some correlation with it, with clinical manifestations of early-onset colorectal cancer or multiple primary tumors, but with a lower rate of outgrowth than *MLH1* or *MSH2* mutations (6). The mechanism of *MLH3* gene mutation on endometrial carcinogenesis may include: i) *MLH3* mutation leads to abnormal MMR function, which is unable to repair DNA replication errors, increasing the rate of gene mutation and promoting tumorigenesis; ii) *MLH3* forms a complex with *MLH1* to participate in DNA repair, and the mutation may disrupt its interaction, leading to defective repair function; iii) *MLH3* defects may make cells unable to trigger apoptosis after DNA damage, leading to survival of abnormal cells and accumulation of oncogenic mutations (10). Patients with *MLH3* mutations by the above mechanisms either exhibit microsatellite instability or are accompanied by somatic mutations. In this case, the patient’s genetic test results showed microsatellite stability without *MLH3* somatic mutation, but it still does not exclude *MLH3* as a possible causative factor for the development of endometrial carcinoma, as in atypical HNPCC.

In the available cohort study of *MLH3* mutations, a family index case was found to have endometrial cancer along with her daughter and thought 80-year-old aunt. In the Database of Genomic Variation and Phenotype in Humans using Ensembl Resource (https://www.deciphergenomics.org/), it was shown that patients with a mutation at the 14q24.3 locus of the *MLH3* gene, showed an endometrial cancer and enhanced tumor susceptibility, which is consistent with the results of previous cohort studies and similar to the results of the present case report. Although neither of the two patients in this case had the *MLH3* somatic mutation, we found that *MLH3* has an impact on the prognosis of endometrial cancer (https://www.proteinatlas.org) (**Supplementary Figure a**). This case report did not have testing done for the mutated locus, but their being a low-risk family, the *MLH3* gene may also serve as a genetic risk factor for low epistasis.

Thus, we suggest that in high-risk families, single-gene susceptibility remains the preferred factor for the development of the disease; however, in low-risk groups, mutations in genetic risk genes with low epistasis that lead to increased susceptibility and synergistic effects with other mutations may also serve as one of the pathogenic mechanisms. This is why we believe that *MLH3* plays a crucial role in the development of endometrial cancer in this low-risk family.

This report identified germline heterozygous mutations in two patients, indicating a potential role for *MLH3* in endometrial carcinogenesis and suggesting the presence of more germline mutations in endometrial cancers. This highlights the need for improved cancer screening. Future comprehensive genetic testing may lead to a better understanding of abnormal expression in germline genes associated with endometrial cancer.

## 4 Conclusion

This report identifies germline heterozygous mutations in two patients, suggesting a potential role for *MLH3* in endometrial carcinogenesis, which may act as a low-risk factor to increase the risk of tumor susceptibility and does not rule out the possibility of synergistic increases in pathogenicity with other genes. This highlights the need for improved cancer screening. Future comprehensive genetic testing may provide a better understanding of the abnormal expression of germline genes associated with endometrial cancer.

## Data Availability

The original contributions presented in the study are included in the article/**Supplementary Material**. Further inquiries can be directed to the corresponding author.
