# Supplementary figures and images for: Follow-up of hereditary endometrial carcinoma caused by MLH3 gene mutation: a case report
Source: Front Oncol. 2025 Apr 28;15:1532908. doi: 10.3389/fonc.2025.1532908 (PMC12066324; doi:10.3389/fonc.2025.1532908)

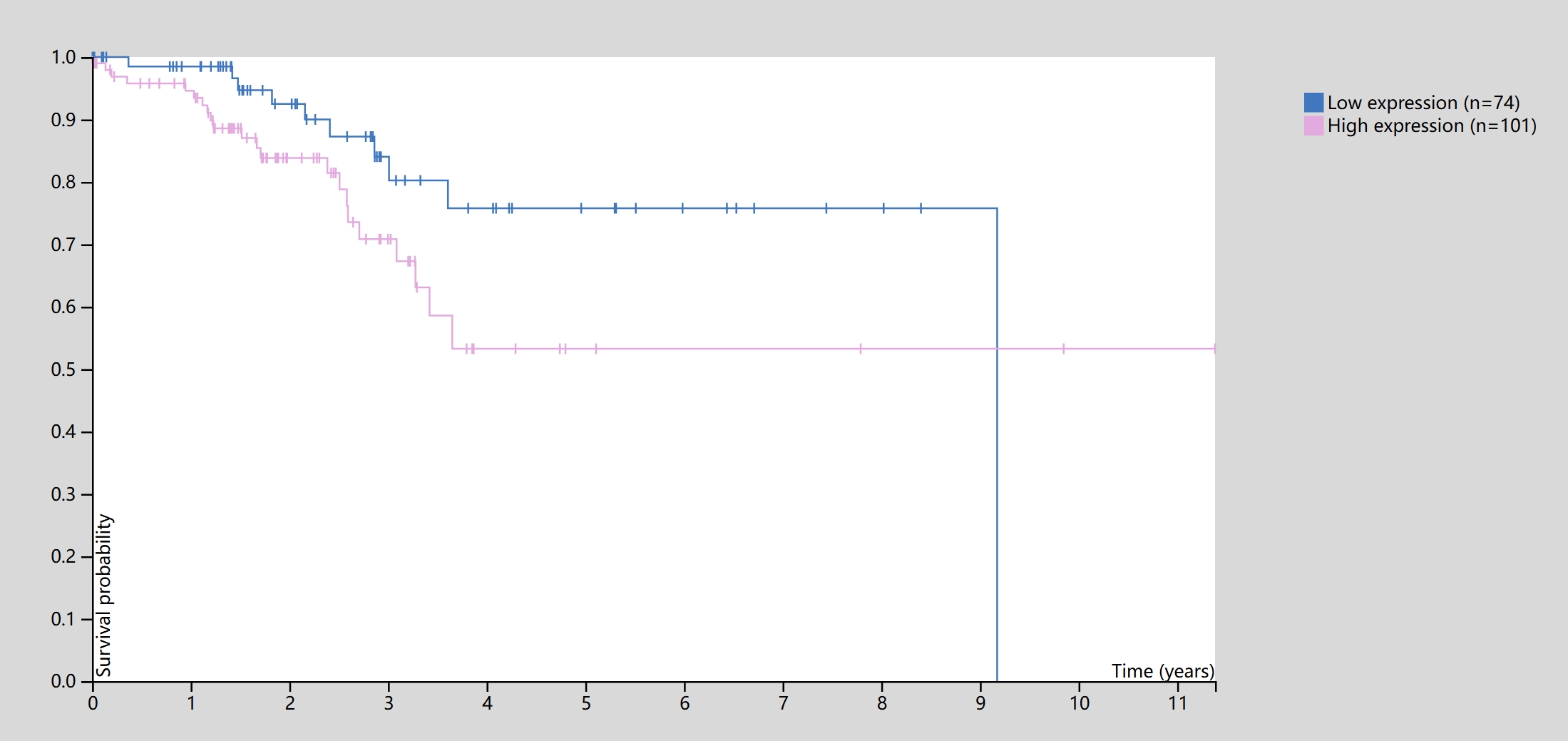

Supplement: Supplementary file 1 [file Image1.jpeg]
